# Retraction: Circular RNA hsa_circ_0056836 functions an oncogenic gene in hepatocellular carcinoma through modulating miR-766-3p/FOSL2 axis

**DOI:** 10.18632/aging.205525

**Published:** 2024-01-14

**Authors:** Zhiqin Li, Yang Liu, Jingya Yan, Qinglei Zeng, Yushu Hu, Hongyan Wang, Hua Li, Juan Li, Zujiang Yu

**Affiliations:** 1 Department of Infectious Disease, The First Affiliated Hospital of Zhengzhou University, Zhengzho 450052, Henan, China

**Keywords:** hepatocellular carcinoma (HCC), circular RNA (circRNA), migration, proliferation, biomarker

**This article has been retracted:** Aging has completed its investigation of this paper. We found multiple instances of internal and external overlap between some of the wound healing assay images used for **Figures 3A** and **5A.** We also found instances of overlap between some of the transwell assay images in **Figures 3C** and **5C**. In addition, numerous small image duplications were found in **Figure 5C**. The authors reported manipulation of **Figures 3** and **5** and falsification of data in **Figure 5**. This represents a major deviation from established scientific standards for publications, and we are therefore retracting this paper. The administration of Zhengzhou University, the institution affiliated with the published Aging article, was notified about this retraction.

